# Antecedents of Vaccine Hesitancy in WEIRD and East Asian Contexts

**DOI:** 10.3389/fpsyg.2021.747721

**Published:** 2021-12-16

**Authors:** Daniel S. Courtney, Ana-Maria Bliuc

**Affiliations:** School of Social Sciences, University of Dundee, Dundee, United Kingdom

**Keywords:** cultural context, WEIRD, anti-vaccine, vaccine hesitancy, East Asia, vaccine attitudes

## Abstract

Following decreasing vaccination rates over the last two decades, understanding the roots of vaccine hesitancy has become a public health priority. Vaccine hesitancy is linked to scientifically unfounded fears around the MMR vaccine and autism which are often fuelled by misinformation spread on social media. To counteract the effects of misinformation about vaccines and in particular the falling vaccination rates, much research has focused on identifying the antecedents of vaccine hesitancy. As antecedents of vaccine hesitancy are contextually dependent, a one-size-fits-all approach is unlikely to be successful in non-WEIRD (Western, Educated, Industrialised, Rich, and Democratic) populations, and even in certain (non-typical) WEIRD sub-populations. Successful interventions to reduce vaccine hesitancy must be based on understanding of the specific context. To identify potential contextual differences in the antecedents of vaccine hesitancy, we review research from three non-WEIRD populations in East Asia, and three WEIRD sub-populations. We find that regardless of the context, mistrust seems to be the key factor leading to vaccine hesitancy. However, the object of mistrust varies across WEIRD and non-WEIRD populations, and across WEIRD subgroups suggesting that effective science communication must be mindful of these differences.

## Introduction

Vaccine hesitancy is defined as “delay in acceptance or refusal of vaccination despite availability of vaccination services” (MacDonald et al., 2015, p. 4161). It is seen as the primary cause of decreasing vaccine rates and resurgences of vaccine-preventable illnesses in many countries (Wise, 2018, 2019; Gardner et al., 2020; Simms et al., 2020). With the COVID-19 pandemic still affecting many parts of the world, combating vaccine hesitancy is particularly important. The roots of vaccine hesitancy are not monolithic across cultures (Hornsey et al., 2018), yet many generalisations are made from studies exclusively conducted on WEIRD (Western, Educated, Industrialised, Rich, and Democratic) populations (Henrich et al., 2010). WEIRD populations tend to differ from non-WEIRD populations in various traits, for example being more individualistic, having more independent self-concepts and being less motivated to conform to the group (Henrich et al., 2010). Amongst non-WEIRD populations, it has been argued that East Asians are the most different to Westerners in this regard (Nisbett, 2005). Even within western countries, group differences exist, for example, liberals in WEIRD countries tend to display more WEIRD cognition than conservatives (Talhelm et al., 2015). Such differences likely impact vaccination attitudes between groups; for example, having higher motivation to conform would likely influence a minority of vaccine hesitant people in a population where vaccination was the norm. This mini-review aims to identify key differences in antecedents of vaccine hesitancy in both subgroups of WEIRD and non-WEIRD (specifically East Asian) populations.

### Anti-vaccination Movements

Opposition to vaccines has existed as long as vaccines (Leask, 2020) - in the 19th Century, anti-Vaccination Leagues, citing personal liberty, opposed smallpox vaccination (Wolfe and Sharp, 2002). Distrust of medicine and fears around efficacy also contributed to opposition (Porter and Porter, 1988). Anti-vaccine sentiments persisted throughout the 20th Century, and gained momentum when British doctor Andrew Wakefield claimed a link between the MMR vaccine and autism (Hussain et al., 2018). The study’s flaws and Wakefield’s monetary conflict of interest resulted in the study’s retraction and Wakefield’s removal from the United Kingdom Medical Registry. Despite the autism-vaccine link being debunked, vaccination rates have continued to fall, leading to rising cases of measles in the United States, United Kingdom, Ireland, and France. Similar stories exist outside the western world. In Japan, after media reports claiming adverse reactions to the HPV vaccine in 2013, the government stopped recommending the vaccine. Vaccination rates dropped from over 70% to below 1%, potentially leading to over 20,000 cases of cervical cancer and 5000 deaths for vaccines missed 2013–2019 (Simms et al., 2020).

## Antecedents of Vaccine Hesitancy

### The Deficit Model of Science Communication

This model, used to explain failures in reducing vaccine hesitancy (Kitta and Goldberg, 2017; Hornsey et al., 2018), proposes that a deficit of knowledge is the main driver of misalignment between scientific consensus and public understanding. As such, the role of science communicators is simply to inform the public of accurate information (Gross, 1994). However, this approach has been found largely ineffective or even to backfire (Hornsey et al., 2018), leading to calls for approaches based on evidence of the root causes of vaccine hesitancy (Kitta and Goldberg, 2017).

### Alliterative Models

The 3Cs model of vaccine hesitancy consists of Confidence, Complacency and Convenience (MacDonald et al., 2015). Confidence refers to trust in vaccines, health professionals who provide them, and vaccine policy makers. Complacency is the belief that vaccines are unnecessary due to the lack of risk perceived from vaccine-preventable diseases. Convenience includes availability, affordability, and accessibility, in addition to barriers to understanding such as language and health literacy. This model was expanded to include a 4th C - Calculation of the risks of vaccination vs. non-vaccination (Betsch et al., 2015), and then a 5th; a sense of Collective responsibility towards one’s community (Betsch et al., 2018). For example, parents without strong pre-existing pro- or anti-vaccine beliefs are likely to engage in Calculation, in particular those who are risk-averse (Betsch et al., 2015, 2018). Extensive searching will likely uncover anti-vaccine information, potentially leading to a falsely perceived equivalence between pro- and anti-vaccine evidence. Therefore, Calculation is predicted to correlate positively with vaccine hesitancy, along with Complacency and Convenience (renamed Constraints in this model). Conversely, Collective responsibility manifests willingness to vaccinate one’s children to help increase herd immunity, and hence correlates negatively with vaccine hesitancy together with Confidence.

It is possible that the 5Cs could be influenced by vaccine-specific issues. For example, following the MMR/autism controversy, confidence in the vaccine decreased, despite complacency towards measles, mumps, and rubella remaining low. Conversely, with the H1N1 vaccine, intention to vaccinate was high before the vaccine was available, but decreased when the virus appeared to be less deadly than first thought, suggesting complacency had set in Velan (2011). Both complacency, especially in the young, and low confidence due to the speed of development of the covid-19 vaccines have been cited as reasons for hesitancy (Mavron, 2021).

### Proximal and Distal Antecedents

The antecedents discussed above can all be described as proximal antecedents, in that they are close to the decision to delay or refuse vaccination in the causal chain of events leading to that decision (Krieger, 2008). Distal antecedents are those further back in the chain, those that lead to people lacking knowledge (e.g., poor science education/communication), or confidence (e.g., conspiracy theories about vaccines). The motivation for this review is to highlight how disparate distal antecedents can lead to similar proximal antecedents of vaccine hesitancy. This matters in combatting vaccine hesitancy as, for example, to instil confidence, it is necessary to understand what causes a lack of confidence.

## Methods

### Search Strategy

The search proceeded in three stages (A breakdown of the search process and papers identified at each stage is included in Supplementary Table 1). Initially, the aim was to use studies from as wide a range of geopolitical contexts as possible, i.e., one from Africa, one from South America, etc. However, after reviewing the studies found in stage one, we found it difficult to assess hesitancy in populations from developing nations, for example in sub-Saharan Africa, as availability of vaccines is a confounding factor. At this stage we discovered differences in hesitancy between White and African Americans, and thus decided to investigate sub-populations of WEIRD countries in the second stage. In the third stage, aiming to capture populations that were non-WEIRD but did not suffer from the confound of vaccine availability, we searched for populations in East Asia, which has highly developed countries which are still considered cognitively and culturally distinct from the west (Nisbett, 2005).

### Inclusion Criteria

After our initial search, we selected 6 articles that complied to our inclusion criteria: 1: empirical studies, 2: peer-reviewed and published recently (2017–2021), on 3a: East-Asian populations, or 3b: differences between demographics within WEIRD populations.

## Findings

The studies non-WEIRD populations studies are: Du et al. (2020); Kwok et al. (2021), and Mizumachi et al. (2021). Those for WEIRD sub-populations are: Freimuth et al. (2017); Hornsey et al. (2020), and Ward et al. (2020). An overview of the studies is presented in Table 1.

**TABLE 1:** Overview of studies.

East Asian populations	*n*	Demographic	Vaccines	Antecedents
Du et al., 2020	2124	Chinese caregivers	Non-specific	3 Cs, scandals
Kwok et al., 2021	1205	Hong Kong nurses	Influenza/COVID-19	5 Cs
Mizumachi et al., 2021	1884	Japanese parents	HPV	Knowledge deficit
**WEIRD sub-populations**				
Freimuth et al., 2017	1643	Black/White United States adults	Influenza	Trust
Hornsey et al., 2020	834	United States voters	MMR/Non-specific	Political leadership
Ward et al., 2020	5018	French citizens (age 18+)	COVID-19	Extreme politics

## Vaccine Hesitancy in East Asian Populations

### Chinese Caregivers

Du et al. (2020) surveyed 2124 caregivers of children under 6 years old from Guangdong, Anhui, and Shaanxi provinces in China, sampled in a cluster process which selected 3–4 communities per province of different social strata. In this sample, 60% expressed some vaccine hesitancy: 3% refused a vaccine for their child, 30.7% delayed a vaccination, and 26.2% had doubts but vaccinated their children. Hesitant caregivers were asked questions about the reasons for their hesitancy, based on the 3 Cs model, and about fears of needles and experiences or information. This final topic is important in the Chinese context due to multiple scandals involving vaccine safety and effectiveness (five since 2005), including in Anhui and Shaanxi provinces. Of those who had heard of the latest scandal (6 months prior to the survey), 61.2% indicated hesitancy, compared to only 50.6% of those who had not. However, the authors include “vaccinated with doubts” in their definition of hesitancy. Using our definition of hesitancy, which only includes delaying or refusing vaccines, this pattern disappears. As such, we can say the scandal increased distrust of vaccines, if not hesitancy.

### Hong Kong Nurses

Kwok et al. (2021) surveyed a convenience sample of 1205 members of the Association of Hong Kong Nursing Staff, during the Coronavirus pandemic of 2020 (March-April), about previous influenza vaccine uptake, intentions towards the COVID-19 vaccine and vaccine hesitancy, as measured by the expanded 5 Cs model. Only 63% of the nurses intended to take the COVID-19 vaccination, argued to be insufficient for herd immunity. The vaccination rate for influenza was 49%, up from 35.6% in a survey taken just 3 years earlier (Kwok et al., 2019). The authors suggest this increased uptake may have been influenced by the pandemic.

Confidence and Collective responsibility were the strongest predictors of both influenza vaccine uptake and COVID-19 vaccination intention. All 5 Cs were significant predictors of COVID-19 vaccination intention and all except Calculation were significant predictors of influenza vaccine uptake. The authors also investigated the relationship between daily case rate and intentions to vaccinate, by tracking the average intention on each day during which the survey was conducted, finding vaccine intentions were higher earlier in their study when cases were higher than towards the end when cases decreased. This suggests the sense of immediate danger from the virus felt by the nurses influenced their intentions to vaccinate.

### Japanese Parents

Mizumachi et al. (2021) surveyed a convenience sample of 1884 Japanese parents visiting paediatric departments of eight hospitals in Nara prefecture about hesitancy towards the HPV vaccine. In addition to questions about the vaccine, they provided accurate information about the HPV vaccine’s safety and efficacy. They found exposure to information about the vaccine increased intentions to vaccinate children from 21.8 to 50.2%. In total, 79.9% of respondents had heard media reports linking the vaccine to adverse effects, but only 33.5% understood the likelihood of the vaccine preventing cervical cancer is higher than of serious adverse reactions. While this near doubling of parents willing to vaccinate after simply reading corrective information can be viewed as support for the deficit model of science communication, nearly half (*N* = 925) of the parents were still unwilling to vaccinate their children. They asked parents unwilling to vaccinate what factors could change their minds. Common responses include (a) communication from healthcare providers (35.1%), (b) media reports of vaccine safety (26.7%), (c) government recommendation (19.5%), and (d) other children’s parents accepting the vaccine (16.8%). Finally, 4.4% indicated no information would change their minds, including 0.2% who would not allow their children any vaccine.

## Vaccine Hesitancy in WEIRD Sub-Populations

### White and African Americans

Freimuth et al. (2017) surveyed a stratified sample of 834 non-Hispanic White and 809 non-Hispanic African Americans, designed to be representative of the United States population, and post-stratification weighted to adjust for over- and under-sampling. They investigated the role of trust in the racial disparity in influenza vaccine rates: 47 and 39%, respectively (Centers for Disease Control and Prevention [CDC], 2016). While the authors found higher vaccination rates in their sample, 53.4 and 44.4%, the magnitude of the racial disparity was similar. The authors focus on generalised trust and specific trust in the influenza vaccine, including questions on trust in information about the vaccine from healthcare professionals and organisations such as the CDC and WHO. African American respondents showed significantly lower trust than Whites on every question except trust in government information on the vaccine. The largest differences were in generalised trust and trust in healthcare professionals. The authors also report trust in the influenza vaccine was lower than for vaccines in general in both groups.

The authors argue the lower trust among African Americans is unsurprising “given historical and contemporary experiences with racism” (p. 76). Research shows African Americans receive lower quality healthcare than Whites in general (Egede, 2006), and for specific issues such as arthritis (Constantinescu et al., 2009) and various cancers (Hershman et al., 2005; Hardy et al., 2009; Fiala and Wildes, 2017). While disparities in insurance coverage exist, these differences in health outcomes persist even when insurance is accounted for (Lillie-Blanton and Hoffman, 2005).

### Republican and Democratic Voters in the 2016 United States Election

Hornsey et al. (2020) conducted two studies using convenience samples following the 2016 United States election to investigate the influence on vaccine hesitancy of having voted for an openly anti-vaccine president, Donald Trump. The first was a survey of voters (*N* = 518) about vaccine concern, political ideology, and conspiratorial beliefs. The second was an experimental design (*N* = 316), comparing the responses of participants to the same questions before and after exposure to Trump’s tweets.

In Study 1, conservatism and conspiracist ideation were significant predictors of hesitancy towards the MMR vaccine and vaccines in general. Trump voters (*N* = 168) were significantly more hesitant than non-Trump voters (*N* = 350). However, when controlled for conservatism and conspiracist ideation, this difference was non-significant. The authors suggest two possible explanations; that those high in conservatism and conspiracist ideation (and thus vaccine hesitancy) tended to vote for Trump, or that Trump influences vaccine attitudes in his supporters.

Study 2 found exposure to Trump’s anti-vaccine tweets increased vaccine hesitancy amongst his supporters. Participants answered the same questions as in Study 1 twice, one week apart. Directly before the second responses, participants were randomly assigned to an experimental group; shown anti-vaccine tweets by Trump, or a control group; shown Trump tweets about golf. Trump voters in the experimental group expressed significantly more vaccine hesitancy after exposure to the tweets. The authors argue while this does not rule out Trump attracting vaccine-hesitant voters, it supports the hypothesis that his statements can influence his supporters’ views on vaccines.

### French Partisans

Ward et al. (2020) surveyed a sample of 5018 French citizens, stratified to be representative of the French population, during April 2020 about their demographics, partisanship, and willingness to accept a COVID-19 vaccine. France is one of the most vaccine-hesitant countries in the world (Ward et al., 2019). Unlike in the United States, where conservatism correlated with vaccine hesitancy, French supporters of both Far-Left and Far-Right parties, and those who did not support any party were more vaccine-hesitant than those who supported more centrist parties.

Partisanship was divided into four categories; Far-Left, Far-Right, Left/Centre/Right, and Green. Respondents who did not feel close to any party were asked to indicate whether or not they voted in the previous election, in 2017. Among Left/Centre/Right supporters 12.5% would “probably” or “certainly” refuse the COVID-19 vaccine. This number was over 20% for all other groups, the highest being non-voters (37.6%) and Far-Right (33.1%) voters, with Far-Left (28.8%), and Green (24.4%) voters in the middle.

The authors suggest two trends in French politics could explain these results: the rise of abstention and the transformation of the political landscape. Voter turnout has decreased in France, with the 2017 election being the first when a majority of voters abstained (Alexandropoulos, 2017). Ward et al. (2020) argue this, combined with vaccine hesitancy among non-partisans shows distrust of institutions. The authors also state the political landscape has shifted in France since 2011, with voters moving away from the traditional centre-left and right parties, towards both the extremes and President Macron’s centrist LaREM. They argue while no political leader had spoken out against the COVID-19 vaccine specifically, voters could see vaccine intention as inherently political, with more extreme parties being more anti-vaccine.

### Integration of Findings Across Studies

We assessed the six studies based on sample size, appropriateness of measures and validity of findings (see Supplementary Table 2). Only two of the studies had any issues; Du et al. (2020) included “vaccinated with doubts” in their measure of hesitancy, without which their finding that knowledge of vaccine scandals increased hesitancy does not hold, while Freimuth et al. (2017) did not analyse the data for the 3Cs, despite this having been collected in this survey and being used in a separate study (Quinn et al., 2019).

An overview of the findings across the six studies is shown in Table 2. Several patterns in the results can be ascertained. First, there is something of a split between the East Asian and WEIRD studies. The researchers in the Asian contexts have all chosen specific populations to study, nurses, and parents/caregivers, whereas in the WEIRD contexts the populations are more general. While distrust is a proximal antecedent of vaccine hesitancy in each study, there is a split between East Asia, where the object of distrust tends to be the vaccines themselves, and the WEIRD contexts where distrust is more towards a vague “Establishment.”

**TABLE 2:** Overview of findings.

Study	Du et al., 2020	Kwok et al., 2021	Mizumachi et al., 2021	Freimuth et al., 2017	Hornsey et al., 2020	Ward et al., 2020
Variables measured	Hesitancy (inc. doubts) 3Cs Reasons	5Cs Flu uptake Covid intentions Work stress	Knowledge of HPV vaccine	Trust: general, vaccines, vaccine process	Conspiracism Gen. vaccine concern MMR vaccine concern Conservatism Voting	Vaccine concern Vaccine intention Party affiliation
Population	Chinese caregivers	Hong Kong nurses	Japanese parents	Black/White United States adults	United States voters	French citizens
Findings	Knowledge of vax scandal increased doubts, but not other hesitancy	5Cs correlate with uptake/intentions	HPV safety unknown (66–71%) Info doubled acceptance	AAs less flu vax (44–53%) Lower in all 3 trust variables	Trump voters higher concern Exposure to tweets + concern	Far-left/right + no party = higher refusal
Object of mistrust	Vaccines in general (medical establishment) Government	Vaccines in general (medical establishment)	Vaccines in general (medical establishment)	Vaccines in general (medical establishment) Institutions (WHO, CDC)	The Establishment	The Establishment
Demographic and other correlates	Age Father/mother Religion Education Income	Age	NA	Age Income Political ideology Perceived racism	Conspiracism Conservatism	Age Income

Where demographic information was collected, correlations with hesitancy were found. However, these correlations did not produce a consistent pattern across the studies. In France (Ward et al., 2020) and the United States (Freimuth et al., 2017) age negatively correlated with distrust, the opposite was true with Hong Kong Nurses (Kwok et al., 2021). Amongst the Chinese caregivers (Du et al., 2020) distrust was higher in 30–35 year olds than those younger, but this pattern did not continue with higher age groups. Education correlated positively with distrust in the Chinese caregivers (Du et al., 2020), but negatively among Americans (Freimuth et al., 2017). Income correlated negatively with distrust amongst French citizens (Ward et al., 2020) and African Americans, but no correlation was found in White Americans (Freimuth et al., 2017) or Chinese caregivers (Du et al., 2020). Finally both studies in the United States (Freimuth et al., 2017; Hornsey et al., 2020) found political conservatism correlated positively with distrust. This overview shows that, while there are some similarities in the findings of the different studies, their heterogeny emphasises the importance of context to understanding vaccine hesitancy.

## Discussion

We examined vaccine hesitancy based on research in six contexts from both WEIRD and non-WEIRD populations. The cultural, political, medical, and informational environment differs in each context. Despite these differences, distrust is a proximal antecedent of vaccine hesitancy in all these contexts. However, the object of this distrust, the distal antecedent, differs with context – that is, the vaccines in the Asian populations, and institutions in the Western populations.

The studies in the three Asian contexts found distrust specifically towards vaccines was the main antecedent of vaccine hesitancy. The causes of vaccine distrust are different in the Chinese and Japanese contexts - distrust in Japan seems to stem from misinformation about the dangers of vaccines, while distrust in China seems driven by accurate information about local vaccine scandals. Kwok et al.’s (2021) study did not specifically focus on the causes or objects of distrust in Hong Kong, but rather on the direct predictors of vaccine uptake and intention to vaccinate. Consistent with the findings from Chinese and Japanese populations, they found the opposite of distrust, i.e., confidence (as trust in vaccines, health professionals, and relevant health authorities) was the strongest predictor of both vaccine uptake and intention.

We found in the Japanese context, the deficit model may be effective in combatting vaccine hesitancy, with roughly a fifth of parents changing their minds based on the information in the survey, and the majority of those who did not indicating a willingness to change their minds following corrective information from the right source. A combination of reintroducing government recommendation of the HPV vaccine, reported along with accurate safety data in the media and better communication from doctors could significantly improve vaccination coverage. However, the history of vaccine scandals in China turns the deficit model on its head. Although vaccine hesitancy in WEIRD populations has been linked to misinformation such as the much debunked MMR-autism link (Jolley and Douglas, 2014), Du et al. (2020) found accurate knowledge of scandals correlated with vaccine hesitancy. Government messaging seems unlikely to improve trust in vaccines, as trust in government has declined recently (Zhao and Hu, 2017).

From the studies in Western contexts, a link between distrust in vaccines and institutions promoting and administering them seems plausible. For African Americans, the distrust was driven by objective disparities in treatment and outcomes between White and African Americans and was directed towards medical professionals and organisations. For Trump supporters and French extremists, the distrust was directed more towards government or a vaguely defined “establishment.” Freimuth et al. (2017) ask about trust in vaccine advice given by relevant institutions, but not specifically about trust in the institutions themselves. However, as vaccines are a medical issue, distrust in advice from medical professionals and organisations suggests distrust in these as sources of information in general. As in the Chinese context, this distrust is not entirely irrational, in particular in the United States where outcome disparities between African and White Americans are well-documented. Ironically, if this type of distrust leads to lower vaccination rates, it is almost certain to further increase disparities in vaccine-preventable diseases.

While Hornsey et al. (2020) do not explicitly draw a link between Trump and distrust, Trump was famously known as the anti-establishment candidate. The correlation between conservatism and vaccine hesitancy could be interpreted as differing from the French context, where both extremes are more hesitant than the centre. However, there was no far-left candidate in the 2016 United States election, Clinton being much more centrist than Trump (Zurcher, 2016). It is possible United States citizens with far-left views are also more vaccine-hesitant than centrists.

### Limitations and Strengths

As a mini review, this paper is limited by the number of studies it was possible to cover. Even within the two categories reviewed (WEIRD sub-populations and East Asian populations) there are populations which are not covered, for example, British minority groups. Furthermore, East Asians are a small segment of the non-WEIRD world, chosen specifically for their unrepresentativeness, in that hesitancy is clearly distinct from unavailability in these countries. As the studies did not use representative samples of their populations, and did not directly ask participants which factors influenced their vaccination decisions, it was not possible to assess which factors were the most prevalent in these populations. The main strength, however, is in showing that even within this limited sample, significant variation exists in distal antecedents of vaccine hesitancy, suggesting that one-size-fits-all approaches to promoting vaccines are unlikely to be the most successful.

## Conclusion

The studies reviewed here suggest the distal antecedents of vaccine hesitancy vary with context, something which must be considered for successful science communication. This comports with a 2015 systematic review which found that “tailored interventions to specific populations and their specific concerns were most effective” (Zurcher, 2016, p. 4184) in increasing vaccine uptake. They found the most successful interventions were directly targeted at specific populations, informed them, increased convenience, legally enforced vaccination and used influential leaders (e.g., religious leaders) to promote vaccination. As this only covers vaccine uptake, it cannot speak directly to the influence these interventions had on trust in vaccines. Vaccine mandates for example, likely increase uptake regardless improved trust, and could even be counterproductive on that front. However, it is likely that any positive effect on uptake due to information and the influence of leaders is due to increases in trust.

In the studies reviewed in our paper, it appears communication of accurate information from government and healthcare providers may be successful in Japan. However, this is unlikely to convince those such as Trump supporters and extreme French voters who are distrustful of institutions. Likewise, African Americans who distrust medical organisations and Chinese who distrust their government are unlikely to heed messages from these sources. If trust does prove to be a common factor in vaccine hesitancy across contexts, having those trusted by members of vaccine-hesitant communities speak in favour of vaccines could be an effective intervention in vaccine promotion.

## Author Contributions

DC conducted the review and wrote the first draft of the manuscript. A-MB contributed to the final draft. Both authors jointly conceived of the theme and direction of the review.

## Conflict of Interest

The authors declare that the research was conducted in the absence of any commercial or financial relationships that could be construed as a potential conflict of interest.

## Publisher’s Note

All claims expressed in this article are solely those of the authors and do not necessarily represent those of their affiliated organizations, or those of the publisher, the editors and the reviewers. Any product that may be evaluated in this article, or claim that may be made by its manufacturer, is not guaranteed or endorsed by the publisher.
