# Supplementary material for: Antecedents of Vaccine Hesitancy in WEIRD and East Asian Contexts
Source: Front Psychol. 2021 Dec 16;12:747721. doi: 10.3389/fpsyg.2021.747721 (PMC8716949; doi:10.3389/fpsyg.2021.747721)
Supplement: Supplementary file 1 [file Table_1.DOCX]

Antecedents of Vaccine Hesitancy in WEIRD and East Asian Contexts

**Table 1.** Stages of search

| **Stage** | **Target** | **Search terms** | **Papers identified** |
| --- | --- | --- | --- |
| 1 | Countries from each continent | “Antecedents of vaccine sceptic attitudes” OR “anti-vaccine” OR “vaccine conspiracy” OR “vaccine hesitancy Japan” OR “vaccine hesitancy Africa” OR “vaccine hesitancy Uganda” OR “vaccine hesitancy Ethiopia” OR + Since 2017 | Bangura et al., 2020 (1)  Al-Mohaithef & Padhi, 2020 (2)  Wagner et al., 2019 (3)  P. R. Ward et al., 2017 (4)  Acheampong et al., 2021 (5)  Carcelen et al., 2021 (6)  Afolabi & Ilesanmi, 2021 (7)  Cooper et al., 2018 (8)  Cooper et al., 2021 (9) |
| 2 | WEIRD sub-populations | "vaccine hesitancy" OR "vaccine sceptic" OR "anti-vaccine" + "African Americans" OR left-wing OR right-wing + Since 2017 | Baumgaertner et al., 2018 (10)  Bodily et al., 2020 (11)  Hornsey et al., 2020 (12)  Quinn et al., 2019 (13)  Freimuth et al., 2017 (14)  Razai et al., 2021 (15)  J. K. Ward et al., 2019 (16)  J. K. Ward et al., 2020 (17) |
| 3 | East Asia | "vaccine hesitancy" OR "vaccine sceptic" OR "anti-vaccine" + Japan OR "Hong Kong" OR China | Simms et al., 2020 (18)  Kwok et al., 2019 (19)  Kwok et al., 2021 (20)  Mizumachi et al., 2021 (21)  Shimizu et al., 2020 (22)  Du et al., 2020 (23)  Wagner et al., 2021 (24) |

**Table 2.** Quality Assessment of Studies

| **Study** | **Research Question** | **Measures appropriate** | **Sample size** | **Interpretations valid** |
| --- | --- | --- | --- | --- |
| Du et al., 2020 | Determinants of VH in China, esp. Changchun Changsheng vaccine incident | Inc. vax w/ doubts in hesitancy | 2124 | Effect of CCVI due to doubt, not delay/refusal |
| Kwok et al., 2021 | Influenza vax uptake  Covid-10 vax intentions  Antecedents | Yes | 1205 | Yes |
| Mizumachi et al., 2021 | Parental HPV vaccine acceptance  Factors that influence | Yes | 1884 | Yes |
| Freimuth et al., 2017 | Determinants of trust in flu vax for African/White Am’s | 3Cs not included | 1643 | Yes |
| Hornsey et al., 2020 | Do Trump’s anti-vax views influence supporters? | Yes | 834 | Yes |
| Ward et al., 2020 | Vax intentions of French voters | Yes | 5018 | Yes |

**References**

1. Bangura JB, Xiao S, Qiu D, Ouyang F, Chen L. Barriers to Childhood Immunization in Sub-Saharan Africa: A Systematic Review. 2020;

2. Al-Mohaithef M, Padhi BK. Determinants of covid-19 vaccine acceptance in saudi arabia: A web-based national survey. J Multidiscip Healthc. 2020;13:1657–63.

3. Wagner AL, Masters NB, Domek GJ, Mathew JL, Sun X, Asturias EJ, et al. Comparisons of vaccine hesitancy across five low- and middle-income countries. Vaccines. 2019;7(4):1–11.

4. Ward PR, Attwell K, Meyer SB, Rokkas P, Leask J. Understanding the perceived logic of care by parents: A qualitative study in Australia. PLoS One. 2017;12(10):1–15.

5. Acheampong T, Akorsikumah EA, Osae-Kwapong J, Khalid M, Appiah A, Amuasi JH. Examining vaccine hesitancy in sub-saharan africa: A survey of the knowledge and attitudes among adults to receive covid-19 vaccines in ghana. Vaccines. 2021;9(8).

6. Carcelen AC, Prosperi C, Mutembo S, Chongwe G, Mwansa FD, Ndubani P, et al. COVID-19 vaccine hesitancy in Zambia: a glimpse at the possible challenges ahead for COVID-19 vaccination rollout in sub-Saharan Africa. Hum Vaccines Immunother [Internet]. 2021;00(00):1–6. Available from: https://doi.org/10.1080/21645515.2021.1948784

7. Afolabi AA, Ilesanmi OS. Dealing with vaccine hesitancy in Africa: The prospective COVID-19 vaccine context. Pan Afr Med J. 2021;38(3):1–7.

8. Cooper S, Betsch C, Sambala EZ, Mchiza N, Wiysonge CS. Vaccine hesitancy–a potential threat to the achievements of vaccination programmes in Africa. Hum Vaccines Immunother [Internet]. 2018;14(10):2355–7. Available from: https://doi.org/10.1080/21645515.2018.1460987

9. Cooper S, van Rooyen H, Wiysonge CS. COVID-19 vaccine hesitancy in South Africa: how can we maximize uptake of COVID-19 vaccines? Expert Rev Vaccines [Internet]. 2021;20(8):921–33. Available from: https://doi.org/10.1080/14760584.2021.1949291

10. Baumgaertner B, Carlisle JE, Justwan F. The influence of political ideology and trust on willingness to vaccinate. PLoS One. 2018;13(1):1–13.

11. Bodily JM, Tsunoda I, Alexander JS. Scientific Evaluation of the Court Evidence Submitted to the 2019 Human Papillomavirus Vaccine Libel Case and Its Decision in Japan. Front Med. 2020;7(July):2019–21.

12. Hornsey MJ, Lobera J, Díaz-Catalán C. Vaccine hesitancy is strongly associated with distrust of conventional medicine, and only weakly associated with trust in alternative medicine. Soc Sci Med [Internet]. 2020;255(April):113019. Available from: https://doi.org/10.1016/j.socscimed.2020.113019

13. Quinn SC, Jamison AM, An J, Hancock GR, Freimuth VS. Measuring vaccine hesitancy, confidence, trust and flu vaccine uptake: Results of a national survey of White and African American adults. Vaccine. 2019;37(9):1168–73.

14. Freimuth VS, Jamison AM, An J, Hancock GR, Quinn SC. Determinants of trust in the flu vaccine for African Americans and Whites. Soc Sci Med. 2017;193:70–9.

15. Razai MS, Osama T, McKechnie DGJ, Majeed A. Covid-19 vaccine hesitancy among ethnic minority groups. BMJ [Internet]. 2021;372:n513. Available from: http://www.ncbi.nlm.nih.gov/pubmed/33637577

16. Ward JK, Peretti-Watel P, Bocquier A, Seror V, Verger P. Vaccine hesitancy and coercion: all eyes on France. Nat Immunol [Internet]. 2019;20(10):1257–9. Available from: http://dx.doi.org/10.1038/s41590-019-0488-9

17. Ward JK, Alleaume C, Peretti-Watel P, Seror V, Cortaredona S, Launay O, et al. The French public’s attitudes to a future COVID-19 vaccine: The politicization of a public health issue. Soc Sci Med. 2020;265:2016–21.

18. Simms KT, Hanley SJB, Smith MA, Keane A, Canfell K. Impact of HPV vaccine hesitancy on cervical cancer in Japan: a modelling study. Lancet Public Heal [Internet]. 2020;5(4):e223–34. Available from: http://dx.doi.org/10.1016/S2468-2667(20)30010-4

19. Kwok KO, Li KK, Lee SS, Chng PHY, Wei VWI, Ismail NH, et al. Multi-centre study on cultural dimensions and perceived attitudes of nurses towards influenza vaccination uptake. J Hosp Infect. 2019;102(3):337–42.

20. Kwok KO, Li KK, WEI WI, Tang A, Wong SYS, Lee SS. Influenza vaccine uptake, COVID-19 vaccination intention and vaccine hesitancy among nurses: A survey. Int J Nurs Stud. 2021;114.

21. Mizumachi K, Aoki H, Kitano T, Onishi T, Takeyama M, Shima M. How to recover lost vaccine acceptance? A multi-center survey on HPV vaccine acceptance in Japan. J Infect Chemother [Internet]. 2021;27(3):445–9. Available from: https://doi.org/10.1016/j.jiac.2020.10.012

22. Shimizu K, Sorano S, Iwai K. Vaccine hesitancy in Japan: Is the country well prepared for Tokyo 2020? Travel Med Infect Dis. 2020;(January).

23. Du F, Chantler T, Francis MR, Sun FY, Zhang X, Han K, et al. The determinants of vaccine hesitancy in China: A cross-sectional study following the Changchun Changsheng vaccine incident. Vaccine [Internet]. 2020;38(47):7464–71. Available from: https://doi.org/10.1016/j.vaccine.2020.09.075

24. Wagner AL, Huang Z, Ren J, Laffoon M, Ji M, Pinckney LC, et al. Vaccine Hesitancy and Concerns About Vaccine Safety and Effectiveness in Shanghai, China. Am J Prev Med [Internet]. 2021;60(1):S77–86. Available from: https://doi.org/10.1016/j.amepre.2020.09.003
